# The Organization of Outreach Work for Vulnerable Patients in General Practice during COVID-19: Results from the Cross-Sectional PRICOV-19 Study in 38 Countries

**DOI:** 10.3390/ijerph20043165

**Published:** 2023-02-10

**Authors:** Esther Van Poel, Claire Collins, Peter Groenewegen, Peter Spreeuwenberg, Gazmend Bojaj, Jonila Gabrani, Christian Mallen, Liubove Murauskiene, Milena Šantrić Milićević, Emmily Schaubroeck, Stefanie Stark, Sara Willems

**Affiliations:** 1 Department of Public Health and Primary Care, Ghent University, 9000 Ghent, Belgium; 2 Research Centre, Irish College of General Practitioners, D02 XR68 Dublin, Ireland; 3 Netherlands Institute for Health Services Research, 3500 BN Utrecht, The Netherlands; 4 Department of Sociology, Department of Human Geography, Utrecht University, 9000 Ghent, Belgium; 5 Management of Health Institutions and Services, Heimerer College, 10000 Prishtina, Kosovo; 6 Faculty of Medicine, University of Basel, 4001 Basel, Switzerland; 7 School of Medicine, Keele University, Keele ST5 5BG, UK; 8 Public Health Department, Faculty of Medicine, Vilnius University, LT-03101 Vilnius, Lithuania; 9 Faculty of Medicine, University of Belgrade, 11000 Belgrade, Serbia; 10 Institute of General Practice, Friedrich-Alexander University Erlangen-Nürnberg (FAU), 91054 Erlangen-Nürnberg, Germany

**Keywords:** primary health care, general practice, outreach work, equity, vulnerable populations, community-oriented primary care, quality of care, PRICOV-19, COVID-19, international comparison

## Abstract

The COVID-19 pandemic disproportionately affected vulnerable populations’ access to health care. By proactively reaching out to them, general practices attempted to prevent the underutilization of their services. This paper examined the association between practice and country characteristics and the organization of outreach work in general practices during COVID-19. Linear mixed model analyses with practices nested in countries were performed on the data of 4982 practices from 38 countries. A 4-item scale on outreach work was constructed as the outcome variable with a reliability of 0.77 and 0.97 at the practice and country level. The results showed that many practices set up outreach work, including extracting at least one list of patients with chronic conditions from their electronic medical record (30.1%); and performing telephone outreach to patients with chronic conditions (62.8%), a psychological vulnerability (35.6%), or possible situation of domestic violence or a child-rearing situation (17.2%). Outreach work was positively related to the availability of an administrative assistant or practice manager (*p* < 0.05) or paramedical support staff (*p* < 0.01). Other practice and country characteristics were not significantly associated with undertaking outreach work. Policy and financial interventions supporting general practices to organize outreach work should focus on the range of personnel available to support such practice activities.

## 1. Introduction

The COVID-19 pandemic became the largest global health crisis in living memory that directly and indirectly increased existing health inequities [1]. Being at risk of experiencing severe COVID-19 was influenced by general health status [2,3], whilst specific populations were disproportionally hit by the impact of public health and social measures, such as isolation, physical distancing, and domestic movement restrictions [4]. Despite their higher need for healthcare, the existing barriers to access to care [5,6,7,8] have been even more pronounced during COVID-19 [9], resulting in care delays with possibly poorer health outcomes in the short and long term [10,11]. 

When available, primary care (PC) forms the backbone of comprehensive care for all people [12]. Many PC experts consider outreach work valuable to prevent the underutilization of PC services [13,14,15,16]. Since its origin in Europe in the early 1980s, this strategy has been characterized by values of community-based, approachable, participative, and mutual respect in supporting hard-to-reach or hidden populations [17]. However, a recent scoping review revealed a large variation in its conceptualization [18]. In this paper, outreach work is defined as proactive, provider-initiated care above and beyond demand-led usual care [19]. The effectiveness of outreach work to ensure the continuity of care and deliver preventive care is extensively demonstrated for several health conditions and population groups. For example, a telephone outreach intervention with student volunteers promoted the social well-being of nursing home residents during COVID-19 [20]. Another telephone-based study increased adherence to colorectal cancer screening among ethnic minorities [21].

Although the merits of outreach work are widely acknowledged [13,22], the implementation in practice is regarded as challenging [13,23,24,25,26]. For example, previous evidence demonstrated that several practice characteristics are decisive in setting up outreach work. A French study reported that teaching general practitioners (GPs) more frequently organized criteria-based initiatives for outreach work during COVID-19 compared to non-teaching GPs [22]. They also found this was more common in large, multidisciplinary practices compared to GPs working in small, monodisciplinary practices [22]. However, the underlying mechanisms about the influence of the status of teaching practice, practice staff size, or multidisciplinarity of the team on outreach work were not further elaborated. Another study demonstrated that practices with an above-average number of ethnic minorities were more community-oriented than those with a below-average number [27]. Community orientation refers to the emphasis on policy to increase the welfare of not only the practice population but also the local community [27]. Thus, undertaking outreach work in GP practices might also be closely linked to the composition of the patient population. 

The organization of outreach work in PC could be hampered by its substantial time investment [13,23,24,25] as practices already face a high burden [28]. Thus, the availability of support within the practice regarding staff and financial resources might be important to manage the workload. Previous research showed that being able to delegate the organization to non-GP staff members was a facilitator [29]. Regarding financial resources, the payment model in practice could stimulate outreach work. GPs working in a capitation or salaried system, with a fixed income per patient or hour worked, might be more likely to engage in outreach initiatives because this does not result in a loss of income [27]. However, the evidence is inconclusive on the impact of being reimbursed or getting financial incentives for organizing outreach work in PC [30,31,32]. 

Moreover, the literature on outreach work is often limited to single-country studies with small sample sizes [33,34,35,36,37]. Yet, one can expect that country or healthcare characteristics also determine the ease of implementing outreach work. During COVID-19, the differences between countries might have increased as the pandemic has put healthcare systems under increased but also varied pressure [38]. For example, the intensity of COVID-19 during the first wave of the pandemic varied among European countries, with a higher number of COVID-19 cases in Luxembourg, Spain, and Ireland compared to Greece, Bulgaria, or Hungary [39]. Thus, the organization of outreach work might also differ according to the burden of the pandemic on the country. A European study demonstrated a large international variation in community orientation among GP practices in the pre-COVID era and highlighted the importance of electronic medical records (EMR) in listing vulnerable patients [27]. Hence, outreach work is expected to be more common in practices from countries with a patient list system than those without an obligatory list system, implying having a defined population to take care of [27].

### Study Aim and Hypotheses

Evidence exists on the effectiveness of outreach work to prevent the underutilization of health care among vulnerable populations. Therefore, outreach work is regarded as an intermediary outcome of high-quality care aiming to achieve health equity. This paper investigated the organization of outreach work in GP practices using the international PRICOV-19 database. More specifically, the variation between countries and GP practices was examined during COVID-19, including the role of practice and country-level characteristics. As GP practices already face many challenges in providing high-quality care, the results will inform policy interventions to support GP practices in the organization of outreach work.

Against the background of previous research, two general hypotheses were created to guide the analysis:

➢ Practice characteristics were related to the organization of outreach work in general practice during COVID-19, including:
  ⚬ Practice structure: practice staff size, being a teaching practice, and patient population composition
  ⚬ Burden and available support in practice (personnel and financial resources)

➢ Country characteristics were related to the organization of outreach work in general practice during COVID-19, including:
  ⚬ Intensity of the COVID-19 pandemic
  ⚬ Availability of a patient list system

## 2. Methods

### 2.1. Study Design and Setting

In the summer of 2020, an international consortium of more than 45 research institutes was formed under the coordination of Ghent University (Belgium) to set up the study to consider how primary care practices were organized during the COVID-19 pandemic (PRICOV-19). This multi-country, cross-sectional study aimed to research how general practices (GP practices) were organized during COVID-19 to guarantee high-quality care, how the task roles changed, how the pandemic impacted the well-being of care providers, and whether differences could be found between types of practices and/or healthcare systems. Data were collected in 37 European countries and Israel [40]. The study protocol and data handling protocols are described in the Data Management Plan registered at Ghent University. 

### 2.2. Measurements

Data were collected using an online self-reported survey among GP practices. The survey was developed at Ghent University in multiple phases, including a pilot study among 159 GP practices in Flanders (Belgium). The survey consisted of 53 questions divided into six topics and was translated into 38 languages following a standard procedure. The Research Electronic Data Capture (REDCap) platform was used to host the survey in all languages, send out invitations to the national samples of practices, and securely store the answers from the participants [41]. More details are described elsewhere [40].

The survey data were supplemented by data about the impact of COVID-19 on the country’s population health [39] and the patient list system in the different healthcare systems. The latter was mainly based on data from the Health System Reviews (HiTs), produced by the European Observatory on Health Systems and Policies [42]. HiTs were used if they were published in 2015 or later. Where this source was unavailable for a country, the country coordinators were consulted (applicable to Bosnia and Herzegovina, Cyprus, Hungary, Iceland, Ireland, Italy, Kosovo*, Sweden, and Turkey).

### 2.3. Sampling and Recruitment

Drawing a randomized sample among all GP practices in the country was preferred over convenience sampling. The survey data were collected between November 2020 and December 2021, except for Belgium, where data were partially collected earlier. Data collection varied between countries from three to 35 weeks. The consortium partner(s) recruited GP practices in each partner country following a pre-defined recruitment procedure. Partners logged all the steps taken in the sampling procedure. PRICOV-19 aimed to sample between 80 and 200 GP practices per country, depending on the number of GP practices. Per practice, one survey was completed, preferably by a GP or staff member familiar with the practice organization. The median value of the response rate across all countries was 22.0%, ranging from less than 10% in, among others, Denmark, France, Latvia, and Sweden, to 90% or higher in Serbia, North Macedonia, Greece, and Bulgaria.

### 2.4. Outcome Variables

Four survey items regarding outreach initiatives were selected as the outcome variables in the analyses. More specifically, GP practices were asked to indicate whether one or more of the following initiatives were undertaken in their practice during the COVID-19 pandemic: (i) a list was compiled from the EMR for at least one group of patients with a chronic condition; (ii) this practice contacted patients with a chronic condition who needed follow-up care; (iii) this practice contacted psychologically vulnerable patients; and (iv) this practice contacted patients with known problems of domestic violence or a problematic child-rearing situation. The original answer options were yes, no, and I do not know. Only the data from practices answering yes or no on at least one of the four selected survey items were included in the analyses. 

Next, a composite variable representing “outreach work” was constructed with values ranging from zero to one. Based on the binary answers on the four survey items (yes = 1, no = 0), the scale value was reached by dividing by the number of items. Therefore, a three-level ecometric modelling approach was used with the four survey items as the lowest level. Compared to aggregating the data from the practice level to the country level, this approach had several advantages by considering the multilayered nature of the data. First, this allowed considering the different sample sizes for the composition of the outcome variable, implying that countries with a smaller sample weighted less heavily for the outcome variable. The second advantage is that ecometric modelling allowed for the potentially different composition of the sample of the participating practices among the countries due to selective non-response. Finally, it was possible to calculate the scale’s reliability on the level of the practice and country [43,44].

### 2.5. Independent Variables: Practice Characteristics

Five characteristics of the practice structure were added to the analyses as explanatory variables: practice staff size, measured by the number of paid staff members working in the practice (irrespective of being part-time or full-time); being a teaching practice for GP trainees (yes or no); and three variables regarding the patient population composition. Therefore, respondents were asked to what extent they felt their patient population was below, approximately, or above the average of practices in their country in terms of treating patients with chronic conditions, patients over the age of 70, patients with low (health) literacy, patients with a migration background with difficulty speaking the local language, patients with financial problems, patients with a psychiatric vulnerability, and patients with little social support or limited informal care. If necessary, the respondent could answer I do not know. Due to high inter-relatedness among the items, only the variables on the patient population composition with a significant relationship with the outcome variable in bivariate analyses were added to the analyses. Thus, the variables on patients with a chronic condition, financial problems, and psychiatric vulnerability were selected with the preservation of the original answer options. 

To consider the additional burden on the practice during COVID-19, a survey item was added in which the respondents needed to indicate whether their responsibilities were increased in practice during COVID-19. The original answer options include a 5-point Likert scale ranging from strongly disagree to strongly agree, and I do not know/not applicable.

The explanatory variables regarding the available support were focused on personnel and financial resources. Regarding personnel, two variables were created, including (i) the availability of an administrative assistant or practice manager, and (ii) the availability of paramedical support staff in practice (referring to the presence of at least one staff member of the disciplines of social work, nursing, or health promotion). The original answer options were yes or no for each item. For the financial resources, the survey item on the main payment system in the practice was recoded into a dichotomous variable indicating whether the practice had a capitation payment model or not. This variable was entered at the practice level as it varied within countries.

### 2.6. Independent Variables: Country Characteristics

In the context of the intensity of the pandemic, the numbers of COVID-19 cases and mortality during the three months before the data collection commenced in each country were added as explanatory variables. Furthermore, data on the availability of a patient list system/patient registration with a GP (yes, no) was separately included as a variable.

### 2.7. Data Analysis

Ghent University was responsible for cleaning all data using SPSS Statistics for Windows, version 28.0 (IBM Corp., Armonk, NY, USA). Continuous variables were presented as median and inter-quartile range (IQR). Categorical variables were shown by numbers and valid percentages. Item non-response in the data was high as many respondents did not complete the survey or ticked an invalid answer option (i.e., I do not know or not applicable). However, to keep many cases in the analyses, dummy variables on missing data and missing value indicators for the explanatory variables were created during the recoding process. First, for creating the dummy variables, a different recoding was performed depending on whether the variable was categorical or numeric. Regarding the categorical independent variables, dummy variables were created for each valid answer option in the original variable (e.g., two for the survey item on the status as a teaching practice) with two answer options: yes = 1 (i.e., all cases having ticked that answer option) or no = 0 (i.e., all cases not having ticked that answer option, supplemented by all missing data). In the case of numeric variables, the responses on the original variable were centered (i.e., original value minus the grand mean value), and all missing data were recoded into zero. In the next step, missing value indicators were created per explanatory variable as dummy variables with, in the yes = 1 category, all cases with missing data on the respective survey item and, in the no = 0 category, all cases with valid answers on this (e.g., all cases having ticked yes or no on the survey item on teaching practices). 

Afterwards, linear mixed model analysis (also known as multi-level analysis, hierarchical models, mixed-effect model, mixed models, or nested data models) was undertaken using MLwiN (version 3.02, Centre for Multilevel Modelling, University of Bristol, UK) as GP practices were nested in countries [43]. For each categorical explanatory variable, all created dummy variables were added to the analyses, except for the reference category (e.g., the dummy variable with all cases indicating their practice is not a teaching practice). Numeric explanatory variables were added centered around zero. In addition, all missing value indicators were added to the analyses to verify their relationship with the outcome variable. Hereby, a non-significant relationship between the missing value indicators and outcome variable implies that the missing data were not related to the outcome and were most probably missing at random (31, 32).

In total, four models were tested stepwise in a two-level random intercept regression model: null model (model I, i.e., empty model without explanatory variables to calculate the clustering of the outcome variable within countries), with added variables on practice structure (model II), the burden and available support in the practice (model III), and country characteristics (model IV) as fixed effects. As only 38 countries were included in the analyses, country-level variables were added one at a time in the linear mixed models. The boundary values for the criterion of statistical significance (*p*, two-sided) were determined at *p* < 0.05 at the practice level and *p* < 0.10 at the country level. 

Afterwards, the same analyses were performed on the cases with complete data (*n* = 3620) as a sensitivity analysis. The multicollinearity among the independent variables was checked beforehand.

## 3. Results

In total, 4982 GP practices from 38 countries were included in the analysis. Table 1 describes the four survey items included in the outcome variable. Less than one-third of the practices (30.1%) extracted a list of at least one group of patients with a chronic condition from their EMR during COVID-19. Respectively, 62.8% and 35.6% of the practices contacted patients with a chronic condition or psychological vulnerability. Less than one-fifth of the practices (17.2%) reached out to patients with a known situation of domestic violence or problematic child-rearing.

**Figure 1:**
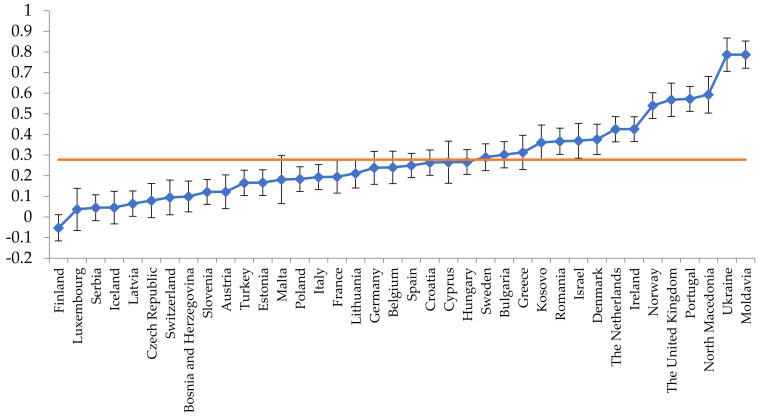
A caterpillar plot for the outcome variable on outreach work per country.

Figure 1 shows a caterpillar plot for the outcome variable on outreach work taking into account the different sample sizes per country. Its mean value was 0.28. The higher the value, the more often outreach work was generally organized in the respective country. The country estimates were also corrected for the covariates in the model. The scale analysis showed good reliability for the outcome variable at the practice (0.77) and country (0.97) level.

Regarding the structure of the participating practices, the median value for the number of paid staff members was 7 (IQR = 3–18) (Table 2). About half of the practices were not teaching practices for GP trainees (52.7%). Based on their subjective perception of the respondent, most practices indicated their practice population include an approximately average number of patients with chronic conditions (54.8%), financial problems (54.1%), or psychiatric vulnerability (62.7%). 

The majority of the respondents (strongly) agreed that overall, their responsibilities had increased in practice during COVID-19 (78.8%, strongly agree: 44.9%). In 57.5% of the practices, an administrative assistant or practice manager was available. Regarding the availability of paramedical support staff, there was a social worker, nurse (assistant), or health promotor in 64.6% of the practices. Approximately half of the practices had a capitation payment system (52.0%).

Table 3 gives an overview of the country characteristics included as explanatory variables in the analyses. Taking into account the country-specific study commencement date, the median value per million inhabitants was 26,879 (IQR = 20,909–35,065) for COVID-19 cases and 488 (IQR = 383–626) for COVID-19 deaths. In 25 countries (65.8%), a patient list system/patient registration with a GP was available. These characteristics for each country are provided as Supplementary Table S1.

Table 4 shows the results of the linear mixed model analyses, including “country” as a random intercept. Model I, showing the null model, or intercept-only model, has an ICC of 47.7, meaning that 47.7% of the variance in outreach work was attributable to the country and 52.3% represented differences among practices. In Model II-III, the variance in outreach work reduced slightly on both levels, which means, in the case of the decreased variance on country-level variance when including practice-level variables, that the distribution of the practice characteristics differs between countries (i.e., composition effect). 

In Model II–III, outreach work was positively related to the availability of an administrative assistant or practice manager (*p* < 0.05) and paramedical support staff (*p* < 0.01). This implies that outreach work was more common in practices with an administrative assistant or practice manager, or practices having a social worker, nurse (assistant), or health promotor available. The other variables were not significantly associated with outreach work. The coefficients of the variables on the practice level were not reported, as these differ only marginally compared to Model III. The COVID-19 cases and deaths in the three months before country-specific data collection commencement or the availability of a patient list system/patient registration with a GP were not significant predictors for outreach work.

As mentioned in the methods section, missing value indicators were added to keep as many cases in the regression models as possible. The analyses showed that there was no significant relationship between any of the missing value indicators and the outcome variable. This implies that the missing data were not related to the outcome and were most probably random. 

Furthermore, sensitivity analyses were performed on the database with complete data to verify the statements (see Supplementary Table S2). These showed that the availability of paramedical support staff was no longer a significant predictor for outreach work. Further analyses revealed this was due to the high number of missing values on the variable regarding the burden in practice. More specifically, this variable was unavailable for 713/4982 cases, excluding for example, all Austrian cases. When removing this variable from the linear mixed model analyses, the same significant predictors for outreach work were found both in the standard as the sensitivity analyses: the availability of an administrative assistant or practice manager (*p* < 0.05) and paramedical support staff (*p* < 0.01) were positively related to outreach work. As the variable on the burden in practice is important against the theoretical background and hypotheses, it was chosen to maintain this variable in the analyses for this paper. 

## 4. Discussion

### 4.1. Main Findings

In this paper, data from the international PRICOV-19 study were used to examine the variation in outreach work in general practice during COVID-19 and its relationship with practice and country characteristics. Hereby, the following two examples of outreach work were selected: using the EMR to select at-risk patients for follow-up care and making proactive telephone calls to vulnerable patients. The results demonstrated that most practices set up at least one initiative for their vulnerable patients and confirmed the hypothesis that the organization of outreach work was related to practice characteristics. However, contrary to the second hypothesis, country characteristics were not associated with outreach work. 

First, comparing the types of outreach work, making proactive telephone calls to patients with a chronic condition was more common among the practices than extracting a list of at-risk patients through the EMR. The rare use of EMRs for outreach work aligns with previous evidence, which could be explained by practices identifying high-risk patients from memory [13], limited knowledge of the EMR system [13], or limited options to summarize patient populations in the EMR software [45]. 

Next, large differences in outreach work between vulnerable groups were found. More precisely, practices more often called patients with a chronic condition than patients with a psychological vulnerability, known domestic violence, or a problematic child-rearing situation. Only 17.2% of the practices proactively reached out to the latter group. However, global leaders have expressed concerns about the alarming numbers of people living in a violent home situation since COVID-19 [46,47]. A possible explanation for why practices only rarely reached out to them could be the difficulty identifying victims of domestic violence [48]. Previous research demonstrated that both patients and GPs experience difficulties discussing this topic: on the one hand, there is evidence among female victims that it takes more than five visits to a healthcare provider before disclosure of abuse [49]; and on the other hand, healthcare providers were barely taught the essential skills to deal with these patients [50,51]. Moreover, a previous study in GP practices demonstrated that only 15% of women with a history of domestic violence had a reference to this in their EMR [52]. This suggests that the patients’ social context was not systematically noted or recorded in the EMR [53]. 

Moreover, the study results confirmed the hypotheses that the setup of outreach work was positively related to practice characteristics. However, this was only applicable to the availability of an administrative assistant or practice manager and paramedical support staff. Our findings align with evidence on having the personnel to whom outreach work could be delegated as an important enabler for its organization [29]. Other studies confirmed that non-GP staff likely to be involved in outreach work usually have a background in social work, nursing, or medical secretary [22,54,55]. 

The variable on the availability of paramedical support staff could also be a proxy for the multidisciplinarity of the practice team. Previous research already pointed out that multidisciplinary teams were at the forefront of implementing innovations in PC services [22]. According to Bouchez, Gautier, Le Breton, Bourgueil and Ramond-Roquin [22], the organization of outreach work was a novelty boosted by COVID-19 and many GPs still considered this as an unusual task. Saint-Lary, et al. [56] also observed a higher ability of multidisciplinary practices to adjust their practice organization to the changing circumstances of COVID-19 compared to monodisciplinary practices. 

Moreover, the country-level characteristics and other elements of the practice structure, burden or availability of support were not related to the setup of outreach work. In terms of the patient population composition, this could be due to the survey item used in PRICOV-19. More specifically, respondents were asked to estimate the proportion of certain vulnerable population groups in their practice compared to the average practices in their country. Therefore, it is necessary to have enough background knowledge of the patient population both in their own practice and other practices of the country. This might be challenging as for example, physicians would often overestimate patients’ income status [57,58]. In contrast to earlier studies, our measure of the additional burden in practice during COVID-19 had no relationship with outreach work. Hereby, respondents were asked to indicate whether their responsibilities increased during COVID-19. This wording could explain the non-significant association with outreach work as previous research already highlighted the high burden on GPs in the pre-COVID era [28], and the used survey item was not centered on outreach work. Moreover, an increased responsibility could be the consequence of outreach work rather than the cause.

### 4.2. Implications for Policy, Practice, and Research

Action is needed to facilitate the identification of vulnerable people as a precondition for outreach work. From a longstanding relationship of trust with patients, GPs are uniquely positioned to pinpoint vulnerable patients in their practice [59]. Therefore, training GPs to assess social determinants of health (SDOH) as possible contributors to poor health outcomes is important. SDOH relate to individual lifestyle factors, social and community networks, living and working circumstances, and macro-level conditions [60]. The study of Herrera, Fiori, Archer-Dyer, Lounsbury and Wylie-Rosett [37] reported on a telephone-based outreach project to train students in these skills during undergraduate medical education. Through a service-based learning approach, medical students gained experience in assessing social needs and making relevant referrals during COVID-19. Given that the participating medical students improved their skills, this initiative may provide a good example for medical education. On the other hand, patients could be encouraged to discuss sensitive topics such as financial problems with their GP through media or GP practice campaigns. 

Previous research demonstrated that the GP’s knowledge of the patient might fail if it is the only method to target patients [22]. Consequently, initiatives are needed to motivate GPs to systematically note or record the patient’s social context in their EMR [61], as this is not routine according to earlier evidence [53]. Moreover, EMR software developers are encouraged to optimize the exchange of information among healthcare professionals through EMR systems and the possibilities to list different patient populations, as such, were often not considered during their development [45]. 

In doing so, the availability of a shared EMR system among healthcare professionals from PC and secondary care may also facilitate an early identification of the vulnerable. For example, in the context of domestic violence, emergency departments and antenatal and mental health services are the main places for early intervention other than GP practices [62,63,64]. However, the rare use of the EMR system for organizing outreach work could also be due to a knowledge gap in its features [13]. Therefore, the implementation of complete training on the EMR system for practice staff is required, rather than relying on “on-the-job” training.

Our study results showed that the availability of personnel is the key factor for organizing outreach work. Thus, policy and financial interventions supporting GP practices to organize outreach work should focus on the range of personnel available to support such practice activities. A close collaboration between policymakers and GPs allows the development of strategies to respond to GPs’ needs to enhance the setup of outreach work. In addition to assessing the patient’s vulnerability, organizing outreach work also requires skills where training can add value [34]. Thus, providing appropriate training for practice staff engaged in outreach work should be prioritized. Working closely with people involved in population management hereby could be inspiring [65].

Finally, future studies on outreach work in GP practices are essential. In this paper, the explanatory variables were selected based on a literature review and preliminary analyses. However, previous evidence suggested other factors that might play a role in the setup of outreach work during COVID-19, such as the characteristics of the GP, having patients who died due to COVID-19 [22], or the organization of outreach work in practice before COVID-19. Given the variation of the outcome variable between countries, other characteristics of the health care system could also play a role in the setup of outreach work. Consequently, future studies need to take a broad perspective on possible explanatory variables from the GP, practice, and country level. 

COVID-19 led to the breakthrough of outreach work in PC. As existing health inequities were increased during COVID-19 and new ones were generated [1], the setup of outreach work will continue to gain importance to be embedded in the daily functioning of the practice. Although this requires resources for its organization in the short term, the potential benefits of alleviating health problems might still be more cost-effective than treating patients with a poor health status due to the postponement of care [55]. Thus, given the many competing demands of practice, research on its cost-effectiveness and sustainability is also crucial. 

### 4.3. Strengths and Limitations

To the best of our knowledge, PRICOV-19 is the largest and most comprehensive study on the organization of general practices during COVID-19. The collaboration with more than 45 research teams led to a large and rich database with about 5000 participants in 38 countries. Despite no funding being available for the PRICOV-19 study, an overall response rate of 22% was achieved. The sample size varied importantly between countries, but the ecometric and multilevel analyses took into account the multilayered nature of the data. Another strength of PRICOV-19 is that the questionnaire was developed and validated in several phases, including a pilot study in Flanders. However, some methodologic and analytic issues might also affect the interpretation and understanding of the study’s findings. 

Firstly, participation in this study was voluntary, so the volunteer bias cannot be ruled out and the response rates varied largely between countries. Country coordinators were encouraged to recruit a randomized national sample to participate in PRICOV-19 as described in the study protocol. However, this recruitment procedure could not be enforced in all countries due to feasibility, for example, because of the lack of a public list of all GP practices in the country and their contact details. It follows that a few country coordinators had to recruit participants on the level of the GP instead of the practice, also because of the lack of funding for the recruitment. 

Moreover, this country-specific recruitment procedure affected the representativeness of the PRICOV-data. Based on the data provided by the country coordinators, it is considered that, overall, the participating practices did not reflect the spectrum of GP practices in the countries involved in terms of the distribution of urbanization (i.e., big city, suburbs, small towns, semi-rural, and rural), size (based on the number of patients), and type (i.e., solo, duo, and group practices based on the number of GPs working in practice). More specifically, practices in small towns and suburbs were generally underrepresented in the PRICOV-19 study, and on the other hand, those located in (semi-) rural areas, large practices (over 10,000 patients), and group practices were overrepresented. However, these conclusions were made for half of the countries based on the perception of the country coordinator as official sources to substantiate these data were often lacking.

Furthermore, the study protocol instructed only one completed survey per practice. Hereto, duplicated cases were checked. Thus, the reliability of the responses might be related to the respondent’s familiarity with the practice organization and outreach work.

Using a self-reported questionnaire always includes the risk that social desirability impacts the respondent’s responses. The researchers have no insight into the actual practice organization and outreach initiatives that were organized. However, the large variation between countries suggests that social desirability did not play a large role (unless there are also large differences between countries in giving socially desirable answers). Next, following the cross-sectional study design, it was impossible to draw causal relationships. In addition, the outcome variable only focused on whether outreach work for several vulnerable groups was organized during COVID-19. How often the selected initiatives were organized in practice was not precisely measured, as well as the follow-up of these initiatives. PRICOV-19 only focused on two types of outreach work in the context of continuity of care, including using the EMR system and proactive telephone calls focusing on three groups: patients with a chronic condition, psychological vulnerability, and known situations of domestic violence or problematic child-rearing. The results could be different when focusing on outreach work for preventive health care or other target groups. 

In addition, the start and length of the data collection period differed among the countries, ranging from three to thirty-five weeks between November 2020 and December 2021. Within and between the participating countries, some GP practices might have had more time to adjust their practice organization and routines to the challenges of the pandemic. PRICOV-19 offered only a snapshot of the practice organization, so the country-specific time frame of the data collection might influence the results. However, to consider the intensity of the pandemic on countries, the COVID-19 cases and mortality rates in the three months before the country-specific start of the data collection were added as explanatory variables. 

## 5. Conclusions

Vulnerable populations in need of care often experience barriers to accessing health care, which has become even worse since COVID-19. The pandemic boosted the organization of outreach work as an innovative approach to prevent the underutilization of PC. PRICOV-19 demonstrated that many GP practices set up outreach work during COVID-19, including extracting a list of patients with chronic conditions who needed follow-up care and telephone calls to patients with a chronic condition, psychological vulnerability, or known problems of domestic violence or child-rearing situation. The results revealed that the setup of outreach work was related to practice characteristics but not to the selected country characteristics. More specifically, outreach work was more common in practices with an administrative assistant or practice manager or paramedical support staff than those without their availability. Therefore, policy and financial interventions supporting GP practices to organize outreach work should focus on the range of personnel available to support such practice activities.

**Note:** * All references to Kosovo, whether the territory, institutions, or population, in this project shall be understood in full compliance with the United Nations Security Council Resolution 1244 and the ICJ Opinion on the Kosovo declaration of independence, without prejudice to the status of Kosovo.

## Figures and Tables

**Table 1:** A description of the survey items included in the outcome variable (*n* = 4982).

Survey Items Included in the Outcome Variable: During COVID-19…	Value	Number	Valid Percent (%)
… a list was compiled from the electronic medical record for at least one group of patients with a chronic condition (*n* = 4618)	Yes	1392	30.1
	No	3226	69.9
… this practice contacted patients with a chronic condition who needed follow-up care (*n* = 4862)	Yes	3051	62.8
	No	1811	37.2
… this practice contacted psychologically vulnerable patients (*n* = 4660)	Yes	1668	35.8
	No	2992	64.2
… this practice contacted patients with previous problems of domestic violence or with a problematic child-rearing situation (*n* = 4338)	Yes	746	17.2
	No	3592	82.8

**Table 2:** A description of the practice characteristics of the participating GP practices (*n* = 4982).

Practice Characteristics	Median (IQR)	Value	Number	Valid Percent (%)
Number of paid staff members (*n* = 4897)	7 (3–18)	-	-	-
Being a teaching practice for GP trainees	-	Yes	2203	47.3
(*n* = 4660)		No	2457	52.7
Patients with chronic conditions ^a^ (*n* = 4851)	-	Below average	226	4.7
		Average	2645	54.8
		Above average	1980	40.8
Patients with financial problems ^a^ (*n* = 4774)	-	Below average	1047	21.9
		Average	2581	54.1
		Above average	1146	24.0
Patients with a psychiatric vulnerability ^a^ (*n* = 4762)	-	Below average	863	18.1
		Average	2986	62.7
		Above average	913	19.2
The responsibilities of the respondent were increased in practice during COVID-19 (*n* = 4269)	-	Strongly disagree	106	2.5
		Disagree	195	4.6
		Neutral	605	14.2
		Agree	1446	33.9
		Strongly agree	1917	44.9
Availability of an administrative assistant or practice manager (*n* = 4979)	-	Yes	2861	57.5
		No	2118	42.5
Availability of paramedical support staff (*n* = 4979)	-	Yes	3214	64.6
		No	1765	35.4
Capitation payment system (*n* = 4879)	-	Yes	2535	52.0
		No	2344	48.0

IQR = inter-quartile range; ^a^ the variables on the patient population composition were based on the subjective perception of the respondent; paramedical support staff refers in this study to the following disciplines: social worker, nurse (assistant), or health promotor.

**Table 3:** A description of the country characteristics of the 38 participating countries.

Country Characteristics	Median (IQR)	Value	Number	Valid Percent (%)
COVID-19 cases per million population during 3 months before survey (*n* = 38)	26,879 (20,909–35,065)	-	-	-
COVID-19 mortality per million population during 3 months before survey (*n* = 38)	488 (383–626)	-	-	-
Availability of a patient list system or patient registration with a GP (*n* = 38)	-	Yes No	25 13	65.8 34.2

IQR = inter-quartile range.

**Table 4:** The results of the linear mixed model analyses with outreach work as outcome variable.

	Model I: Empty Model Coefficient (SE)	Model II: Practice Structure Coefficient (SE)	Model III: Burden and Availability of Support Coefficient (SE)	Model IV: Country Characteristics Coefficient (SE) ^a^
*Fixed part*				
Constant	0.357 (0.033) ***	0.344 (0.034) ***	0.322 (0.035) ***	
Practice staff size				
Number of paid staff members		−0.000 (0.000)	−0.000 (0.000)	
GP trainee teaching practice (ref. no)				
Being a teaching practice: yes		0.012 (0.007) *p* = 0.087	0.007 (0.007)	
Patient population composition (ref. approximately average)				
Patients with chronic conditions: below average		0.001 (0.015)	0.001 (0.015)	
Patients with chronic conditions: above average		0.005 (0.007)	0.005 (0.007)	
Patients with financial problems: below average		0.008 (0.008)	0.009 (0.008)	
Patients with financial problems: above average		0.001 (0.009)	0.001 (0.009)	
Patients with a psychiatric vulnerability: below average		0.011 (0.009)	0.012 (0.009)	
Patients with a psychiatric vulnerability: above average		0.010 (0.009)	0.009 (0.009)	
Burden				
Increased responsibilities during COVID-19			0.006 (0.003) *p* = 0.076	
Availability of support (ref. no)				
Availability of administrative assistant or practice manager: yes			0.017 (0.008) *	
Availability of paramedical support staff: yes			0.024 (0.009) **	
Capitation payment model: yes			−0.009 (0.010)	
Intensity of COVID-19 in the 3 months before country-specific study commencement				
COVID-19 cases per million population				−0.000 (0.000)
COVID-19 mortality per million population				0.000 (0.000)
Patient list system (ref. no)				
Availability of patient list system/ patient registration with a GP: yes				0.067 (0.069)
*Random part*				
Country variance	0.042 (0.010)	0.041 (0.010)	0.041 (0.010)	
Practice variance	0.046 (0.001)	0.046 (0.001)	0.046 (0.001)	
ICC (%)	47.7	47.1	47.1	

* *p* < 0.05; ** *p* < 0.01; *** *p* < 0.001; SE = standard error; ICC = intra-class correlation; ^a^ the explanatory variables on country level have been added one by one due to the relatively small number of countries; coefficients of practice level variables are not reported—they differ only marginally from those in Model III.

## Data Availability

The anonymized data is held at Ghent University and all raw data that could lead to the identification of the respondents were permanently removed. Reasonable request is required to access non-identifiable data by users who are external to the research teams involved. Access will be subject to a data transfer agreement and following approval from the principal investigator of the study.

## Supplementary Materials

The following supporting information can be downloaded at: https://www.mdpi.com/article/10.3390/ijerph20043165/s1, Supplementary Table S1: An overview of the country characteristics; Supplementary Table S2: The results of the linear mixed model analyses with outreach work as outcome variable on the database with complete data.
