# Supplementary material for: The Organization of Outreach Work for Vulnerable Patients in General Practice during COVID-19: Results from the Cross-Sectional PRICOV-19 Study in 38 Countries
Source: Int J Environ Res Public Health. 2023 Feb 10;20(4):3165. doi: 10.3390/ijerph20043165 (PMC9960761; doi:10.3390/ijerph20043165)
Supplement: Supplementary file 1 [file ijerph-20-03165-s001.zip › ijerph-2148936-supplementary.pdf]

Supplementary Material

Supplementary Table S1: An overview of the country characteristics

Country	Per million population: Confirmed COVID-19 cases in 3 months before data collection commenced	Per million population: COVID-19 deaths in 3 months before data collection commenced	Availability of a patient list system/registration with a GP
Austria	33674	729	No
Belgium	42284	580	No
Bosnia and Herzegovina	20735	632	Yes
Bulgaria	23878	1123	Yes
Croatia	27349	898	Yes
Cyprus	27199	204	Yes
Czech Rep	46452	734	No
Denmark	22233	261	Yes
Estonia	7480	41	Yes
Finland	3023	11	Yes
France	22642	488	No
Germany	20039	556	No
Greece	11568	429	No
Hungary	42623	1553	Yes
Iceland	3015	46	Yes
Ireland	27001	279	Yes
Israel	23467	205	No
Italy	29648	633	Yes
Kosovo*	19909	396	No
Latvia	20909	320	Yes
Lithuania	62027	986	Yes
Luxemburg	11537	27	No
Malta	22711	393	No
Moldova	20681	407	Yes
Netherlands	36082	383	Yes
North Macedonia	28624	955	No
Norway	6297	53	Yes
Poland	26879	637	Yes
Portugal	33171	485	Yes
Romania	20220	400	Yes
Serbia	50478	465	Yes
Slovenia	35065	626	Yes
Spain	33318	480	Yes
Sweden	39030	605	Yes
Switzerland	25708	557	No
Turkey	23901	172	Yes
Ukraine	14452	346	No
United Kingdom	8521	307	Yes

Supplementary Material 2: The results of the linear mixed model analyses with outreach work as outcome variable on the database with complete data

	Model I: empty model Coefficient (SE)	Model II: practice structure Coefficient (SE)	Model III: burden and availability of support Coefficient (SE)	Model IV: Country characteristics Coefficient (SE)[a]
Fixed part				
Constant	0.363 (0.034)***	0.346 (0.035)***	0.335 (0.036)***	
Practice staff size				
Number of paid staff members		0.000 (0.000)	0.000 (0.000)	
GP trainee teaching practice (ref. no)				
Being a teaching practice: yes		0.011 (0.008)	0.008 (0.008)	
Patient population composition (ref. approximately average)				
Patients with chronic conditions: below average		0.017 (0.018)	0.018 (0.018)	
Patients with chronic conditions: above average		0.008 (0.008)	0.007 (0.008)	
Patients with financial problems: below average		0.010 (0.010)	0.010 (0.010)	
Patients with financial problems: above average		0.005 (0.010)	0.005 (0.010)	
Patients with a psychiatric vulnerability: below average		0.008 (0.011)	0.008 (0.011)	
Patients with a psychiatric vulnerability: above average		0.013 (0.010)	0.012 (0.010)	
Burden				
Increased responsibilities since COVID-19			0.006 (0.004)	
Availability of support (ref. no)				
Availability of administrative assistant or practice manager: yes			0.018 (0.009)*	
Availability of paramedical support staff: yes			0.008 (0.011)	
Capitation payment model: yes			-0.006 (0.011)	
Intensity of COVID-19 in 3 months before country-specific study commencement				
COVID-19 cases per million population				-0.000 (0.000)
COVID-19 mortality per million population				0.000 (0.000)
Patient list system (ref. no)				
Availability of patient list system/ patient registration with a GP: yes				0.052 (0.073)
Random part				
Country variance	0.043 (0.010)	0.042 (0.010)	0.042 (0.010)	
Practice variance	0.046 (0.001)	0.046 (0.001)	0.046 (0.001)	
ICC (%)	48.3	47.7	47.7	

* $p<0.05$; ** $p<0.01$; *** $p<0.001$; ICC= intra-class correlation; [a]The explanatory variables on country level have been added one by one due to the relatively small number of countries; coefficients of practice level variables are not reported – they differ only marginally from those in Model III.
